# Blocked Randomization with Randomly Selected Block Sizes

**DOI:** 10.3390/ijerph8010015

**Published:** 2010-12-23

**Authors:** Jimmy Efird

**Affiliations:** Center for Health Disparities Research and the Department of Public Health, Brody School of Medicine, East Carolina University, Physicians Quadrangle, Greenville, NC 27858, USA; E-Mail: jimmy.efird@stanfordalumni.org; Tel.: +1-650-248-8282

**Keywords:** blocked randomization, random block sizes, randomized clinical trial

## Abstract

When planning a randomized clinical trial, careful consideration must be given to how participants are selected for various arms of a study. Selection and accidental bias may occur when participants are not assigned to study groups with equal probability. A simple random allocation scheme is a process by which each participant has equal likelihood of being assigned to treatment versus referent groups. However, by chance an unequal number of individuals may be assigned to each arm of the study and thus decrease the power to detect statistically significant differences between groups. Block randomization is a commonly used technique in clinical trial design to reduce bias and achieve balance in the allocation of participants to treatment arms, especially when the sample size is small. This method increases the probability that each arm will contain an equal number of individuals by sequencing participant assignments by block. Yet still, the allocation process may be predictable, for example, when the investigator is not blind and the block size is fixed. This paper provides an overview of blocked randomization and illustrates how to avoid selection bias by using random block sizes.

## 1. Introduction

The purpose of randomization is to achieve balance with respect to known and unknown risk factors in the allocation of participants to treatment arms in a study [1,2]. A premise of basic statistical tests of significance is that underlying observations are independently and identically distributed. The stochastic assignment of participants helps to satisfy this requirement. It also allows the investigator to determine whether observed differences between groups are due to the agent being studied or chance.

By probability, a simple randomization scheme may allocate a different number of participants to each study group. This may reduce the power of a statistical procedure to reject the null hypothesis as statistical power is maximized for equal sample sizes [3]. Additionally, an imbalance of treatment groups within confounding factors may occur. This is especially true for small sample sizes. Confounding distorts the statistical validity of statistical inferences about cause and effect. The failure to control for confounding may inflate type 1 error and erroneously lead to the conclusion that a putative risk factor is causally associated with the outcome under study (*i.e.*, false positive finding). A chance run of participants to a particular study group also may occur under a simple randomization scenario. This can lead to bias, for example, if the initial participants in the trial are healthier than the later ones [1]. Blocked randomization offers a simple means to achieve balance between study arms and to reduce the opportunity for bias and confounding.

## 2. Methodology

Block randomization works by randomizing participants within blocks such that an equal number are assigned to each treatment. For example, given a block size of 4, there are 6 possible ways to equally assign participants to a block. Allocation proceeds by randomly selecting one of the orderings and assigning the next block of participants to study groups according to the specified sequence. Note that repeat blocks may occur when the total sample size is greater than the block size times the number of possible orderings. Furthermore, the block size must be divisible by the number of study groups.

A disadvantage of block randomization is that the allocation of participants may be predictable and result in selection bias when the study groups are unmasked. That is, the treatment assignment that has so far occurred least often in the block likely will be the next chosen [4]. Selection bias may be reduced by using random block sizes and keeping the investigator blind to the size of each block.

### 2.1. Example

**Figure 1:**
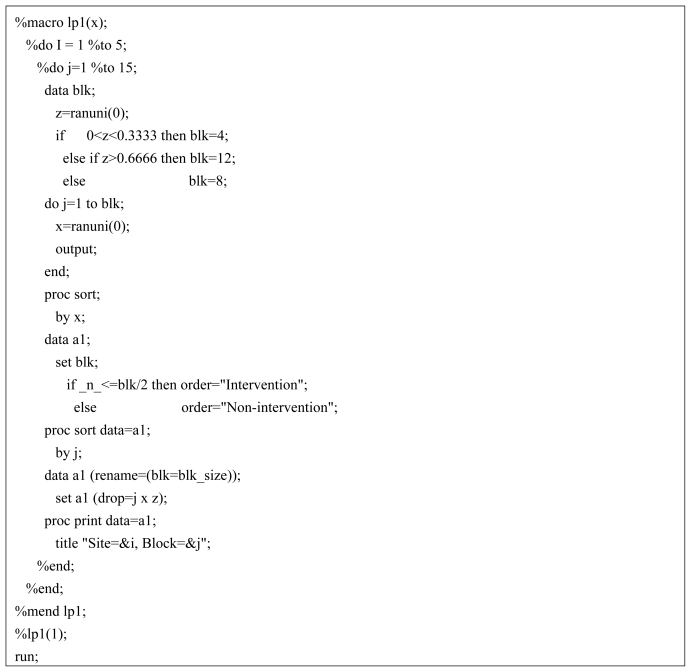
SAS algorithm to perform blocked randomization with random block sizes.

An investigator wishes to compare a family-based educational intervention for childhood weight loss with a standard individual-base program. A planned enrollment of 250 participants, 50 per study site, is to be randomly assigned to the two intervention arms. Below, a computer algorithm written in SAS^®^ (Cary, NC) is presented for performing a block randomization with randomly selected block sizes of 4, 8 and 12 (Figure 1). The macro generates 15 randomized block allocations each for 5 study sites. A greater number of blocks are created than is necessary in the event that the investigator continues enrollment beyond the initially planned sample size. For example, expanded enrollment might occur due to a greater than anticipated attrition rate.

**Figure 2:**
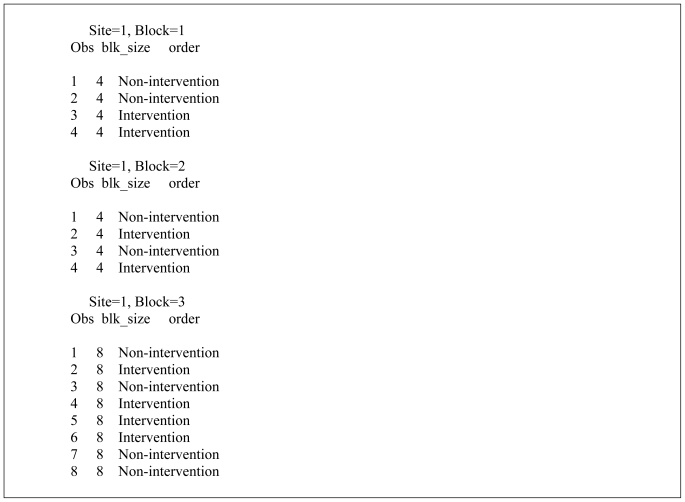
Example output from the SAS algorithm.

The macro works by invoking the ranuni function to equally partition the number of blocks according to a uniform distribution. When the number within the parenthesis of the ranuni function equals zero the seed is determined by the computer system clock. Thus, a different set of block allocations occur each time the macro is executed. Changing the number to a positive integer will assure that the same block allocation is generated during subsequent use of the macro. After the block size is randomly determined the macro efficiently allocates treatment assignment equally within blocks by sorting on the looping index variable. Although the macro only generates 3 randomly selected block sizes the code may be easily modified to increase this number by further partitioning the uniform assignment space. Similarly, the number of study sites and blocks may be increased or decreased by changing the upper range of the two program do-loops. The output of the SAS algorithm corresponding to the first 3 blocks for Site 1 is shown in Figure 2. For example, Block = 1 randomizes 4 participants, with the first two assigned to “Non-intervention” and the last two assigned to “Intervention”.

## 3. Discussion

A key advantage of blocked randomization is that treatment groups will be equal in size and will tend to be uniformly distributed by key outcome-related characteristics. Typically, smaller block sizes will lead to more balanced groups by time than larger block sizes. However, a small block size increases the risk that the allocation process may be predictable, especially if the assignment is open or there is a chance for unmasking of the treatment assignment. For example, certain immunosuppressive agents change color when exposed to light. This may inadvertently expose the identity of the compound in a clinical trial if the comparator compound is not light sensitive. Unmasking also may be intentional in the case of a physician chemically analyzing a patient’s blood to determine the identity of the randomized drug.

Using a large block size will help protect against the investigator predicting the treatment sequence. However, if one treatment occurs with greater frequency at the beginning of a block, a mid-block inequality can occur if there is an interim analysis or the study is terminated midway through a block. Alternatively, keeping block sizes small and using random sequences of block sizes can ameliorate this problem. Another option is to use larger random block sizes but offset the chance of initial treatment runs within a block by allocating participants using a biased coin approach [4]. In a simple trial consisting of a single treatment and referent group, this method probabilistically assigns participants within a block to the treatment arm depending on the assignment balance of participants thus far randomized to the treatment arm. For example, if a participant to be randomized is in a category which has K more treatments (t) than referents (r) already assigned, then assignment to the treatment and referent group will be made with probability t = q, (r = p), t = ½ (r = ½), and t = p, (r = q) contingent on whether K is greater than, equal to, or less than zero (where p ≥ q, p + q = 1). Although the latter strategy may distort the randomization process by decreasing the probability of long runs, the resulting bias may be acceptable if it prevents mid-block inequality and controls the predictability of treatment assignment. Under certain minimax conditions, the random coin approach has been shown to be superior to complete randomization for minimizing accidental bias (e.g., a type of bias that occurs when the randomization scheme does not achieve balance on outcome-related covariates) [4]. A key advantage of the open source algorithm provided in this paper, and comparable algorithms available in programming languages such as R [5], is that the underlying code may be modified to accommodate the random coin technique and other balancing strategies yet to be implemented in standard statistical packages.

The number of participants assigned to each treatment group will be equal when all the blocks are the same size and the overall study sample size is a multiple of the block size. Furthermore, in the case of unequal block sizes, balance is guaranteed if all treatment assignments are made within the final block [1]. However, when random block sizes are used in a multi-site study, the sample size may vary by site but on average will be similar.

The advantage of using random block sizes to reduce selection bias is only observed when assignments can be determined with certainty [1]. That is, when the assignment is not known with certainty but rather is just more probable, then there is no advantage to using random block sizes. The best protection against selection bias is to blind both the ordering of blocks and their respective size. Furthermore, the use of random block sizes is not necessary in an unmasked trial if participants have been randomized as a block rather than individually according to their entry into the study, as the former will completely eliminate selection bias.

The necessity to take into account blocking in the statistical analysis of the data, including when the block sizes are randomly chosen, depends on whether an intrablock correlation exists [1]. A non-zero intrablock correlation may occur, for example, when the characteristics and responses for a participant change according to their entry time into the study. If the process is homogeneous the intrablock correlation will equal zero and blocking may be ignored in the analysis. However, variance estimates must be appropriately adjusted when intrablock correlation is present [6]. The presence of missing data within blocks also can potentially complicate the validity of statistical analysis. For example, special analytic techniques may be needed when the missing data is related to treatment effects or occurs in some other non random manner [1,3]. However, datasets with missing-at-random observations may be analyzed by simply excluding the affected blocks. When possible, measures should be implemented to minimize missing values as their presence will reduce the power of statistical procedures.

Significant treatment imbalances and accidental bias typically do not occur in large blinded trials, especially if randomization can be performed at the onset of the study. However, when treatment assignment is open and sample size is small than a block randomization procedure with randomly chosen block sizes may help maintain balance of treatment assignment and reduce the potential for selection bias.

## References

[1] Matts J Lachin J Properties of permuted-block randomization in clinical trials Control Clin. Trials 1988 9 327 344 3203524 10.1016/0197-2456(88)90047-5

[2] Hewitt C Torgerson D Domanski M McKinlay S Randomization: What is it and how to do it Successful Randomized Trials Wolters Kluwer Philadelphia, PA, USA 2009 Chapter 3 27

[3] Lachin J Properties of simple randomization in clinical trials Control Clin. Trials 1988 9 312 326 3203523 10.1016/0197-2456(88)90046-3

[4] Efron B Forcing a sequential experiment to be balanced Biometrika 1971 58 403

[5] Harrell F Block Randomization with Random Block Sizes Available online: http://biostat.mc.vanderbilt.edu/wiki/Main/BlockRandomizationWithRandomBlockSizes (accessed on 5 September 2010).

[6] White H A heteroskedasticity-consistent covariance matrix estimator and a direct test for heteroskedasticity Econometrica 1980 48 817 838

